# Combined expert-in-the-loop—random forest multiclass segmentation U-net based artificial intelligence model: evaluation of non-small cell lung cancer in fibrotic and non-fibrotic microenvironments

**DOI:** 10.1186/s12967-024-05394-2

**Published:** 2024-07-08

**Authors:** Anjali Saqi, Yucheng Liu, Michelle Garlin Politis, Mary Salvatore, Sachin Jambawalikar

**Affiliations:** 1 Department of Pathology and Cell Biology, Columbia University Irving Medical Center, 630 West 168th Street, New York, NY VC14-215, 10032 USA; 2 Department of Radiation Physics, Atlantic Health System, New Jersey, NJ USA; 3 Department of Radiology, Columbia University Irving Medical Center, New York, NY USA

## Abstract

**Background:**

The tumor microenvironment (TME) plays a key role in lung cancer initiation, proliferation, invasion, and metastasis. Artificial intelligence (AI) methods could potentially accelerate TME analysis. The aims of this study were to (1) assess the feasibility of using hematoxylin and eosin (H&E)-stained whole slide images (WSI) to develop an AI model for evaluating the TME and (2) to characterize the TME of adenocarcinoma (ADCA) and squamous cell carcinoma (SCCA) in fibrotic and non-fibrotic lung.

**Methods:**

The cohort was derived from chest CT scans of patients presenting with lung neoplasms, with and without background fibrosis. WSI images were generated from slides of all 76 available pathology cases with ADCA (*n* = 53) or SCCA (*n* = 23) in fibrotic (*n* = 47) or non-fibrotic (*n* = 29) lung. Detailed ground-truth annotations, including of stroma (i.e., fibrosis, vessels, inflammation), necrosis and background, were performed on WSI and optimized via an expert-in-the-loop (EITL) iterative procedure using a lightweight [random forest (RF)] classifier. A convolution neural network (CNN)-based model was used to achieve tissue-level multiclass segmentation. The model was trained on 25 annotated WSI from 13 cases of ADCA and SCCA within and without fibrosis and then applied to the 76-case cohort. The TME analysis included tumor stroma ratio (TSR), tumor fibrosis ratio (TFR), tumor inflammation ratio (TIR), tumor vessel ratio (TVR), tumor necrosis ratio (TNR), and tumor background ratio (TBR).

**Results:**

The model’s overall classification for precision, sensitivity, and F1-score were 94%, 90%, and 91%, respectively. Statistically significant differences were noted in TSR (*p* = 0.041) and TFR (*p* = 0.001) between fibrotic and non-fibrotic ADCA. Within fibrotic lung, statistically significant differences were present in TFR (*p* = 0.039), TIR (*p* = 0.003), TVR (*p* = 0.041), TNR (*p* = 0.0003), and TBR (*p* = 0.020) between ADCA and SCCA.

**Conclusion:**

The combined EITL—RF CNN model using only H&E WSI can facilitate multiclass evaluation and quantification of the TME. There are significant differences in the TME of ADCA and SCCA present within or without background fibrosis. Future studies are needed to determine the significance of TME on prognosis and treatment.

**Supplementary Information:**

The online version contains supplementary material available at 10.1186/s12967-024-05394-2.

## Background

Lung cancer is the leading cause of cancer-related death in the United States [1] and worldwide [2]. Hematoxylin and eosin (H&E)-stained slides have served as the pillar for lung cancer diagnosis. Over the years, a greater understanding of lung cancer and expanding repertoire of ancillary studies—histochemical, immunohistochemical and molecular diagnostics—have served to complement the established H&E stain. Lung cancer management currently integrates morphological features, ancillary studies and staging, factoring in variables like tumor size, pleural invasion, lymph node involvement and metastasis. This comprehensive approach provides predictive and prognostic information for effective lung cancer management.

Continuously emerging evidence demonstrates that additional factors like the tumor microenvironment (TME) influence tumor development and impact prognosis [3, 4]. For example, multiple studies report an increased incidence of lung cancer in the setting of underlying interstitial lung disease, which corresponds to the microenvironment [5–9]. Recent radiomics studies suggest that pulmonary fibrosis, which corresponds microscopically to TME, observed on chest computed tomography (CT) imaging predisposes to an increased risk for developing lung cancer [10]. On a cellular level, evidence shows that normal fibroblasts, primary precursors of carcinoma-associated fibroblasts and a component of TME, play an important role in carcinogenesis, tumor progression, and angiogenesis through their interaction with other stromal cells [11]. Moreover, the tumor stroma ratio (TSR) derived from TME is an independent prognostic determinant of tumor proliferation, invasion, and metastasis in various types of lung cancers, including non-small cell lung cancer (NSCLC) [11–15].

Despite acknowledging its significant impact, including its potential predictive capabilities for treatment responses to immune checkpoint inhibitors and as a biomarker, TME is currently neither a constituent of pathology staging nor integral to management decisions. The reasons for this disconnect may include delay in translating scientific research into clinical practice, lack of sufficient direct evidence-based studies and limited access to efficient, accessible, and cost-effective quantitative tools that incorporate TME into pathological evaluation.

More recently, digitized whole slide images (WSI) have become available for research and clinical use paving the path for integrating computer-aided diagnosis (CAD) algorithms to assist with arduous tasks otherwise performed by pathologists [16]. This has coincided with the continuous development of and accessibility to robust hardware, enhanced infrastructure speed, open-source software, and artificial intelligence (AI) platforms with convolutional neural networks (CNN) that permit deep learning analysis in pathology [17].

The objectives of this study were twofold: (1) to evaluate the feasibility of developing a model based on WSI using CNN-based artificial intelligence methods for TME analysis and (2) to compare TME in adenocarcinomas (ADCA) and squamous cell carcinomas (SCCA) that developed in the presence or absence of background lung fibrosis observed on CT.

## Methods

### Datasets

Following IRB approval, a retrospective search of chest CT scans was performed as described previously [18]. Cases with pulmonary nodules were classified based on the presence or absence of surrounding fibrotic environment—a nodule was deemed to be within a fibrotic environment if it was at least partially surrounded by fibrosis, and without fibrosis if there was complete lack of surrounding fibrosis, respectively. (Supplementary Fig. 1) When fibrosis was present, usual interstitial pneumonia was the most common pattern. (Table 1)

**Table 1:** Imaging, demographics, available smoking status, specimen subtypes, and pathology diagnoses

Lung Environment (Chest CT)	Fibrotic Lung	Non-fibrotic Lung	Overall
	47	29	76
Airways-centered fibrosis	6	N/A	6
Combined pulmonary fibrosis and emphysema	2	N/A	2
Nonspecific interstitial pneumonia	4	N/A	4
Radiation fibrosis	2	N/A	2
Sarcoid	2	N/A	2
Usual interstitial pneumonia	25	N/A	25
Undetermined	6	N/A	6
Gender			
Male	32	13	45
Female	15	16	31
Age			
50–59		2	2
60–69	7	4	11
70–79	17	12	29
80–89	13	8	21
90 and above	10	3	13
Smoking Status (Available)			
Smoker	11	12	23
Non-smoker	0	6	6
Pathology Diagnosis			
Adenocarcinoma	28	25	53
Squamous cell carcinoma	19	4	23
Specimen subtypes			
Resection	16	14	30
Biopsy	31	15	46
Dominant Histology in Resections			
Adenocarcinoma Resections	12	14	26
Well differentiated*	0	0	0
Moderately differentiated**	12	11	23
Poorly differentiated***	0	3	3
Squamous cell carcinoma	4	0	4

N/A: Not applicable. *Well differentiated represents predominant lepidic pattern. **Moderately differentiated represents predominant acinar or papillary patterns. ***Poorly differentiated represents predominant solid or micro-papillary patterns

Subsequently, a search for pathologically confirmed diagnosis of the radiographically identified nodules was performed. The study cohort was restricted to cases diagnosed as ADCA and SCCA, and where H&E slides of resections or core biopsies were available.

### Preprocessing

All tumor samples underwent formalin-fixation and paraffin embedding (FFPE), were cut at 4–5 microns, and stained with H&E. The slides were then scanned at 40x magnification using Leica Aperio AT2.

The scanned images consisted of foreground (H&E-stained tissue) and background (empty space), with the latter being considerably more extensive in core biopsy relative to surgical resection samples. In order to improve the data analysis efficiency and focus only on areas with relevant information, the foreground was extracted from the background using a thresholding foreground segmentation algorithm implemented in CLAM [19]. The segmented foreground samples were cropped into $512 pixel\times 512 pixel$ tiles. Depending on the size of the segmented foreground tissues, the number of tiles per slide ranged from hundreds to thousands.

To address stain color intensity variations of WSI, which can affect the performance of CAD systems [20], stain normalization was performed using mean and variance optical density normalization before model training.

### Expert-in-the-loop—random forest and multiclass tissue segmentation

**Fig. 1:**
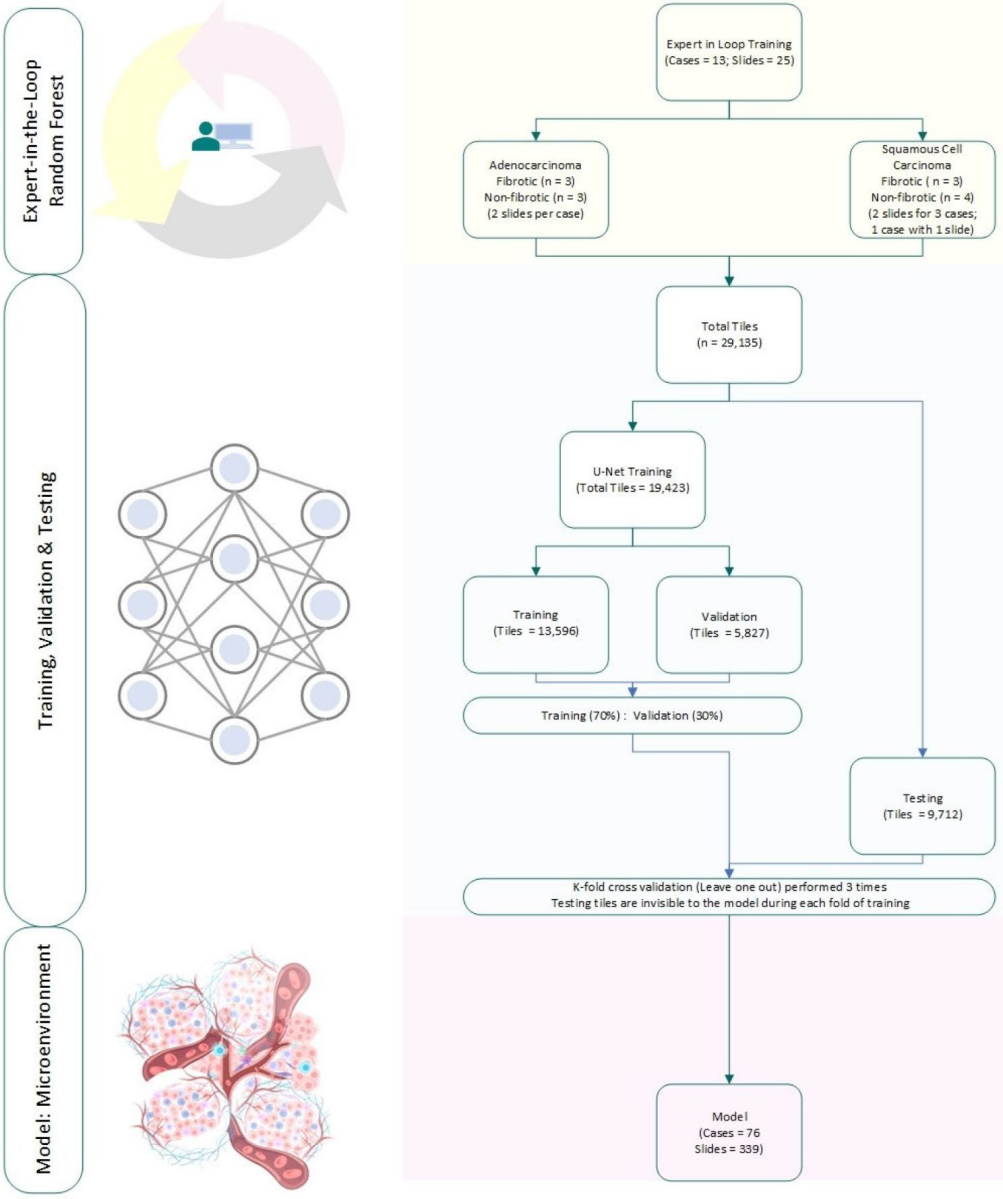
“Expert-in-the-Loop” Workflow: The segmentation model is guided by a pulmonary pathologist. After the initial annotations, a lightweight classifier (i.e., random forest) is applied to generate the whole slide image (WSI) segmentation in real-time. The pathologist reviews the segmented WSI and includes additional annotations based on the results. Note: The analysis is conducted on a tile (not patient) level

Annotations were carried out by a board-certified pathologist on 25 WSI images from 13 individual cases. This included 6 ADCA (3 each in fibrotic and non-fibrotic lung) and 7 SCCA cases (3 in fibrotic and 4 in non-fibrotic lung). All of these were derived from resections except 4 biopsies from non-fibrotic SCCA. To maintain focus on the TME, the analysis was limited to the tumor bed by encircling its border. This yielded 29,135 total tiles that were divided among training, validation and testing without overlap. More specifically, two thirds (19,423) of the tiles were used for training and validation [13,596 (70%) tiles and 5,827 (30%)] and the remaining one third (9,712) tiles were used for testing. (Fig. 1)

QuPath [21], an open-source platform for analysis of 2-dimensional digitized WSIs, was utilized to annotate 10 distinct categories—carcinoma (either ADCA or SCCA), fibrosis (elastotic, dense, loose), vessels, inflammation (macrophages, lymphoplasmacytic), necrosis, and normal tissue (defined as background in this study). Stroma was defined as the summation of fibrosis, inflammation and vessels.

To yield high-quality tissue segmentation with a limited dataset, an “expert-in-the-loop” (EITL) supervised learning workflow was adopted to enhance tissue annotations in the training set and combined with a random forest (RF) algorithm using QuPath. (Fig. 1) RF, a lightweight machine-learning algorithm consisting of 50 classification trees [22], enabled an interactive multiclass segmentation process within a span of 30- to 60-seconds training time and generated a full slide annotation on an individual WSI level. The pathologist continuously reviewed the whole slide multiclass segmentation results as generated by the RF algorithm and performed additional annotations on the weaker performing categories. This EITL-RF model with an iterative review-revise process was repeated ~ 3 to 5 times until the segmentation result visually attained > 80% accuracy, or no notable improvements were observed.

In order to define the region of interest, the tumor border was distinguished from the background by utilizing the RF algorithm, which classified the annotation classes into two binary categories – tumor and non-tumor.

Following the annotation process, the foreground H&E containing areas were segmented into $512 pixel\times 512 pixel$ tiles from the highest resolution (0.25 $\mu m/pixel$) and subsequently downsampled by a factor of 2. Each tile was verified to meet a glass threshold of 30%, thereby ensuring the tile contained sufficient H&E tissue.

A deep-learning CNN model was then trained using the tiles for multiclass segmentation. To achieve this, the U-Net architecture was chosen for multiple reasons. Firstly, U-Net is a well-explored and stable model that has been successfully applied in medical imaging for single class and multiclass segmentation tasks. Secondly, it is particularly suited for smaller size datasets, which is often the case with pathology WSI data [23]. For the U-Net model training, 13 annotated cases (25 WSIs) were used with a training-validation ratio of 70%-30%. The trained model was then applied on the entire 76 cases cohort (339 WSIs) for full slide segmentation and TME analysis (Fig. 1).

### Tumor microenvironment metrics

TME analysis was performed on four subgroups (1) ADCA in fibrotic lung tissue, (2) ADCA in non-fibrotic lung tissue, (3) SCCA in fibrotic lung tissue, and (4) SCCA in non-fibrotic lung tissue.

Quantitative assessment of the TME segmentation results was performed within the pathologist-outlined tumor border using previously defined metrics [tumor stroma ratio (TSR), tumor necrosis ratio (TNR), and tumor inflammation ratio (TIR)] [24, 25]) and new metrics [tumor fibrosis ratio (TFR), tumor vessel ratio (TVR) and tumor background ratio (TBR) ], where background corresponds to non-neoplastic and non-fibrotic lung. Equations 1–6

1 $$TSR = \frac{Stroma}{Stroma + Tumor}\times 100\%$$

2 $$TFR = \frac{Fibrosis}{Fibrosis + Tumor}\times 100\%$$

3 $$TIR = \frac{Inflammation}{Inflammation + Tumor}\times 100\%$$

4 $$TVR = \frac{Vessels}{Vessels + Tumor}\times 100\%$$

5 $$TNR = \frac{Necrosis}{Necrosis + Tumor}\times 100\%$$

6 $$TBR = \frac{Background}{Background + Tumor}\times 100\%$$

### Evaluation

**Fig. 2:**
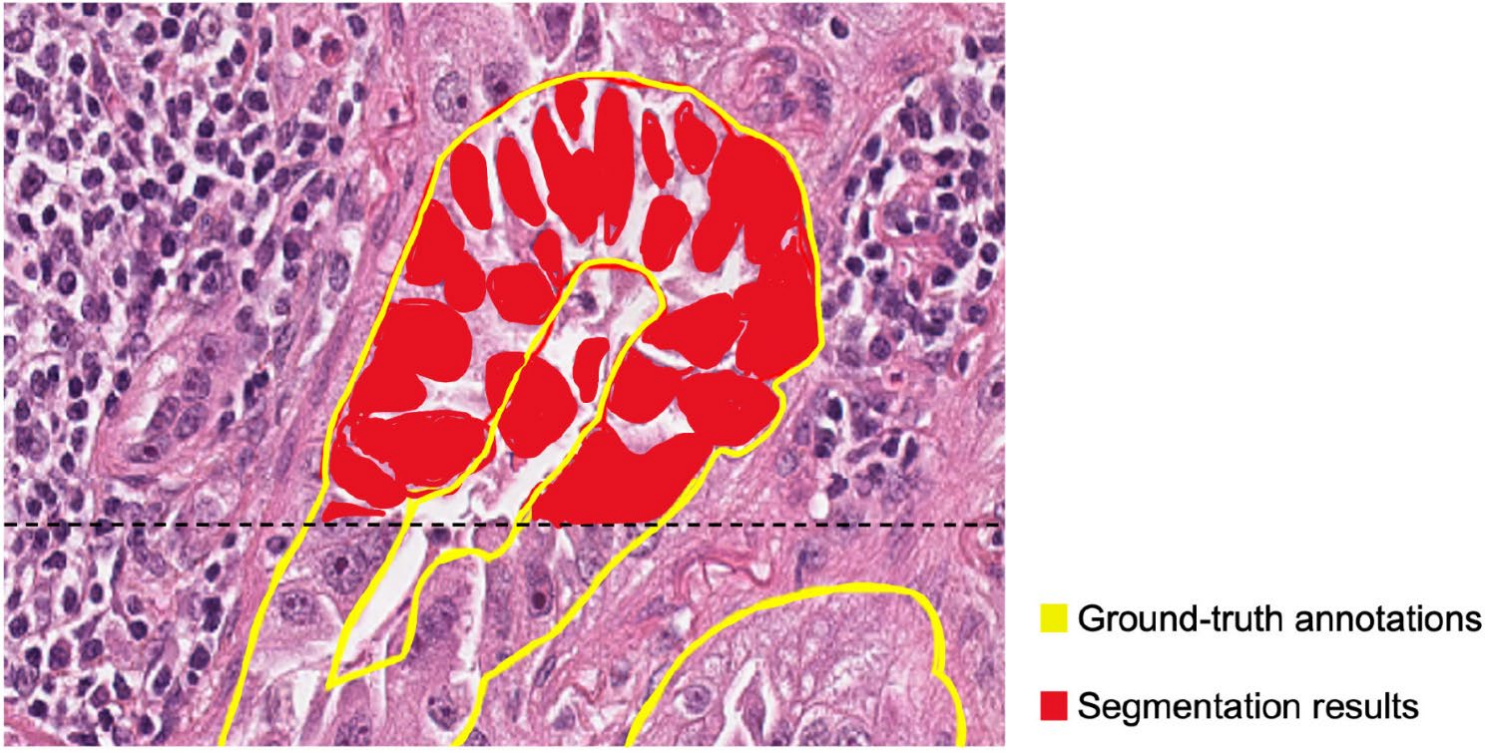
Dice score: An example of adenocarcinoma (ADCA) with ground-truth annotations and auto-segmentation results (only annotations for ADCA and auto-segmentation results above the black line are shown for clarity). The auto-segmentation result is hidden below the dotted black line for better visualization of cancer cells. The pathologist’s ground-truth annotations within the yellow contours are at a cell-cluster level. The auto-segmentation is generated at an individual cell level as demonstrated in red. Due to the scale difference and contour mismatch, the Dice score used to evaluate segmentation results may yield incorrectly low scores

To assess the performance of the segmentation model, a 3-fold cross-validation with a leave-one-out strategy was implemented to evaluate the trained model across the entire cohort. Given that the ground-truth annotations were created at a cell-cluster level and the auto-segmentation was generated at an individual cell level, conventional metrics, such as the Dice score for evaluating segmentation results, would yield incorrectly low scores due to contours mismatch. (Fig. 2) Though evaluating a segmentation model, the goal was to accurately classify WSI tissue, hence metrics commonly used for classification tasks were employed to evaluate model performance for multiclass classification of each tile. Small tiles size of $100 {\mu m}^{2}$was extracted according to the ground-truth annotations. If multiple tissues were segmented within a single tile, the tile’s classification was determined by the tissue with the greatest coverage. Each tile was then compared with the corresponding auto-segmented tile to compute precision (Eq. 7), recall (sensitivity) (Eq. 8), F1-score (Eq. 9), and the confusion matrix. Precision, sensitivity, and F1-score were chosen as classification performance metrics, particularly in the context of multiclass classification problem. Due to the fixed tile size and the fixed threshold design, interpreting ROC (Receiver Operating Characteristic) curves and the associated AUC (Area Under the Curve) becomes more complex. Rather, the confusion matrix permits direct quantification and understanding of the model’s performance as true positives, false positives, true negatives, and false negatives, offering better comprehension of its predictive power and limitations. This level of detail is essential for informed decision making in our pathology application of TME analysis of lung fibrosis and cancer.

7 $$Precision = \frac{TP}{TP+ FP}$$

8 $$Recall \left(Sensitivity\right) = \frac{TP }{TP+FN}$$

9 $$F1-score = 2\times \frac{Recall \times Precision}{Recall + Precision}$$

TP: true positive; TN: true negative; FP: false positive; FN: false negative

### Correlation with doubling times

The doubling time (DT) of carcinomas was calculated when *≥* 2 CT scans were available. Using an online DT calculator for growth rate of a lesion or a mass [26], the diameter and date of the first and second examinations were entered, and the DT in days was generated as described previously [18, 27].

A comparative analysis of DT, ADCA and SCCA was conducted. Available DTs were correlated with TSR, TFR, TIR, TVR, and TNR.

### Statistical analysis

Data are presented as mean ± SD or median (25–75% IQR), based on their distribution. Figures 5A and 5B (Supplementary Table 1A and 1B) Two-tailed p-values below 0.05 were considered statistically significant. Comparisons of cancer TME ratios between fibrotic and non-fibrotic lung tissues, as well as between fibrotic and non-fibrotic tissues in ADCA and SCCA, were conducted using unpaired t-tests for normally distributed data and Wilcoxon rank-sum tests for non-normally distributed data. Additionally, a sub-analysis of ADCA (categorized by short and long DT) and SCCA in fibrotic lung tissue was performed using the same statistical methods as appropriate.

## Results

### Study cohort

A total of 76 cases from 45 men and 31 women fulfilled the study criteria; the mean ages were 80 and 78 years in the fibrotic and non-fibrotic cohorts, respectively. These included 53 ADCA and 23 SCCA present in fibrotic (*n* = 47) or non-fibrotic (*n* = 29) backgrounds from 30 resections and 46 biopsies. (Table 1)) The 26 adenocarcinoma resections were either moderately differentiated (*n* = 23, predominant acinar or papillary patterns) or poorly differentiated (*n* = 3, predominant solid or micropapillary patterns). The biopsies were not categorized but imaging of all adenocarcinomas had a solid component.

### Tissue segmentation

**Fig. 3:**
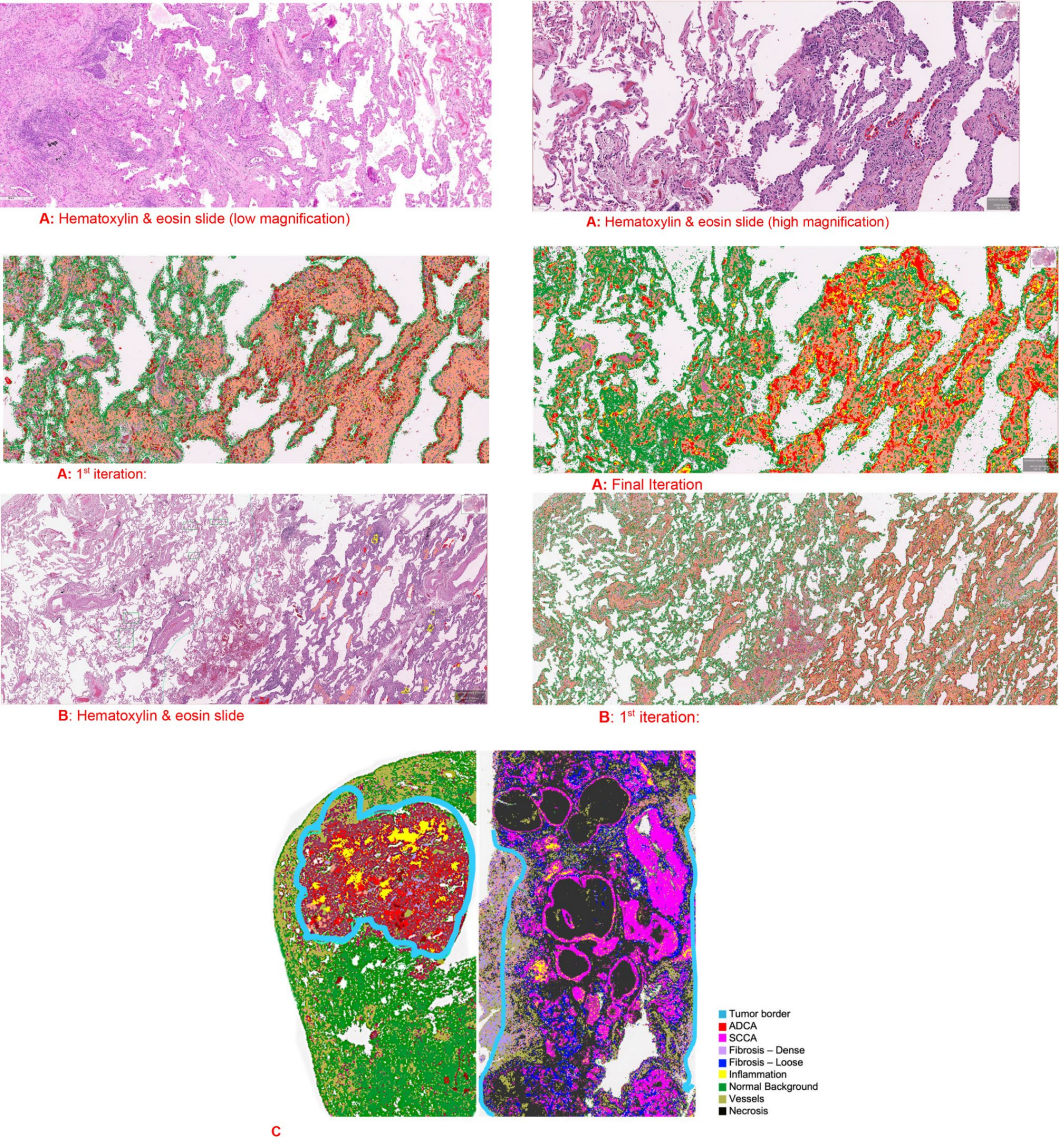
Multiple Iterations **A** and **B**: Two examples of challenging lepidic pattern regions in adenocarcinoma comparing the classification results from the first and final iterations. These demonstrate notable differences in classification of normal lung (green), adenocarcinoma (red) and inflammation (yellow) between the first and final iterations. The initial iterations misclassified the relatively bland-appearing cells of the lepidic pattern as normal; the classification is improved in the final iteration utilizing the EITL-RF annotation process. Carcinoma in Non-fibrotic and Fibrotic Lung **C**: Example cases demonstrate the auto-segmentation results and the tumor border demarcation (light blue). The case on the left is an adenocarcinoma (red) in a non-fibrotic lung environment showing inflammation (yellow). The case on the right is squamous cell carcinoma (pink) with necrosis (black) and fibrosis (lilac and blue) in a fibrotic lung environment

There were $211\pm 85$ average annotations per WSI. The EITL-RF annotation process resulted in an overall 11% increase in classification accuracy. The number of annotations was approximately 2 times greater in the final (cancer cell and fibrosis annotations doubled) versus initial iterations. The EITL-RF process was especially useful in adenocarcinomas with subregions showing relatively subtle differences between the adenocarcinoma and adjacent non-neoplastic lung. For example, some lepidic pattern regions had small neoplastic cells, low-grade cytology and relatively preserved architecture requiring a greater number of annotations to distinguish from other non-neoplastic elements. (Fig. 3)

**Fig. 4:**
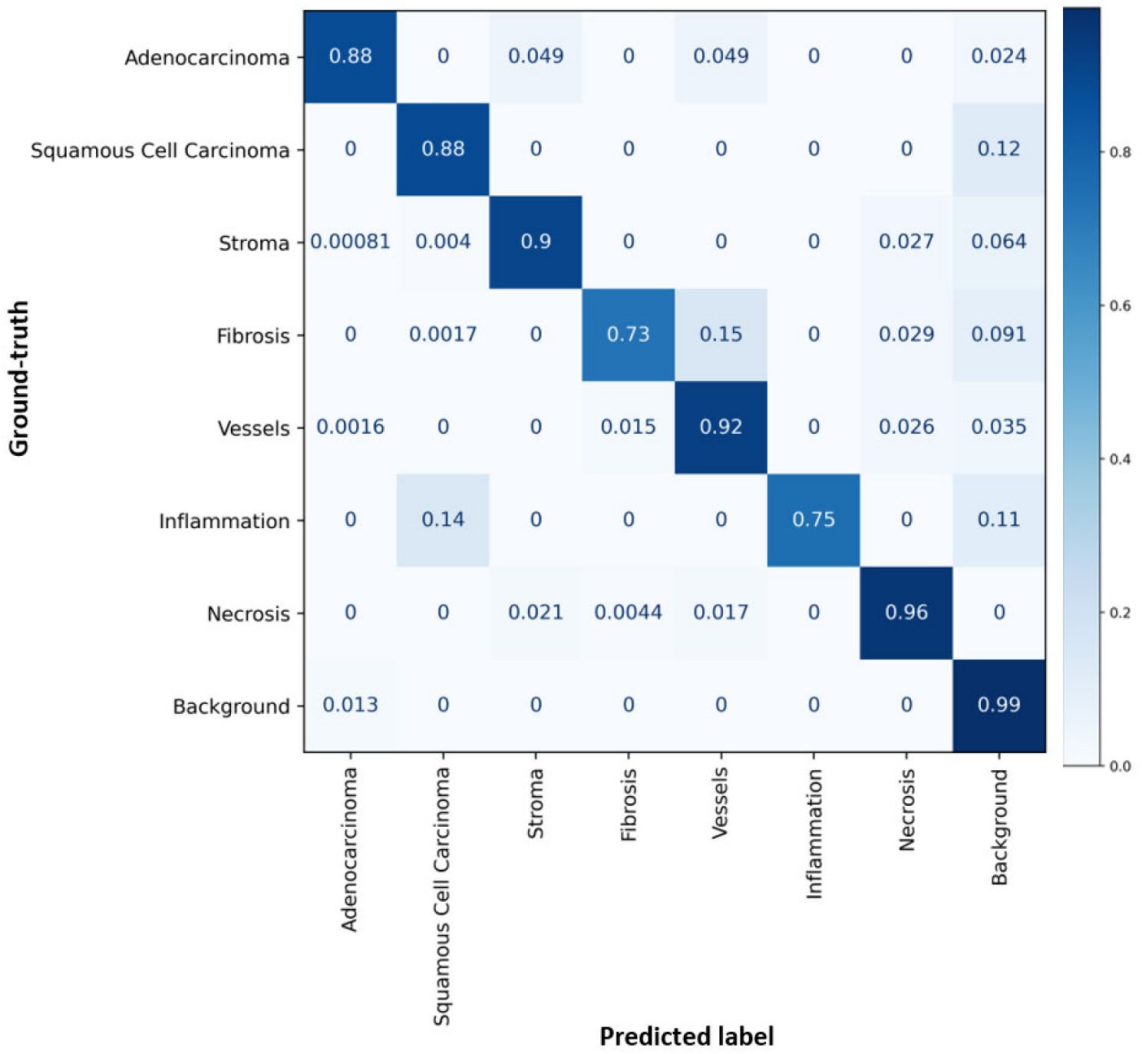
Confusion Matrix: Multiclass auto-segmentation model performance

This process resulted in an EITL-RF auto-segmentation model for tissue analysis. To train and evaluate the model’s performance, a dataset containing a total of 29,135 image tiles was utilized. The model architecture employed a U-Net framework and consisted of an encoder-decoder structure, incorporating four layers of 2D convolutional blocks with a kernel size of 3 × 3. To enhance the model’s learning capabilities, leaky rectified linear units (LeakyReLU) and batch normalization techniques were incorporated. These components helped in capturing and preserving important features during the segmentation process. To optimize memory usage, the feature channels within the model were carefully organized. Specifically, the channels were arranged in a sequence of 32, 64, 128, 256, and 512, following the approach proposed by Oskal et al. in 2019 [28]. This arrangement allowed for efficient utilization of computational resources without compromising the model’s performance. The multiclass tile classification model was evaluated using standard metrics, resulting in excellent performance with an overall precision, sensitivity, and F1-score of 0.94, 0.90, and 0.91, respectively. (Table 2) For a detailed visualization of the auto-segmentation model’s performance, please refer to Fig. 4, which presents the confusion matrix. (Fig. 4) Overall, the segmentation model, employing the U-Net architecture and optimized feature channel arrangement, demonstrated robust performance in accurately segmenting tissue regions in our dataset.

**Table 2:** Multiclass precision, sensitivity and F1

Classes	Precision	Sensitivity (Recall)	F1-Score
Adenocarcinoma	0.96	0.88	0.92
Squamous	0.94	0.88	0.91
Stroma	0.95	0.90	0.90
Fibrosis	0.98	0.73	0.90
Vessels	0.84	0.92	0.93
Inflammation	1.00	0.75	0.86
Necrosis	0.93	0.96	0.96
Background Normal	0.63	0.99	0.77
Weighted Average	0.94	0.90	0.91

### Tumor microenvironment analysis

**Fig. 5:**
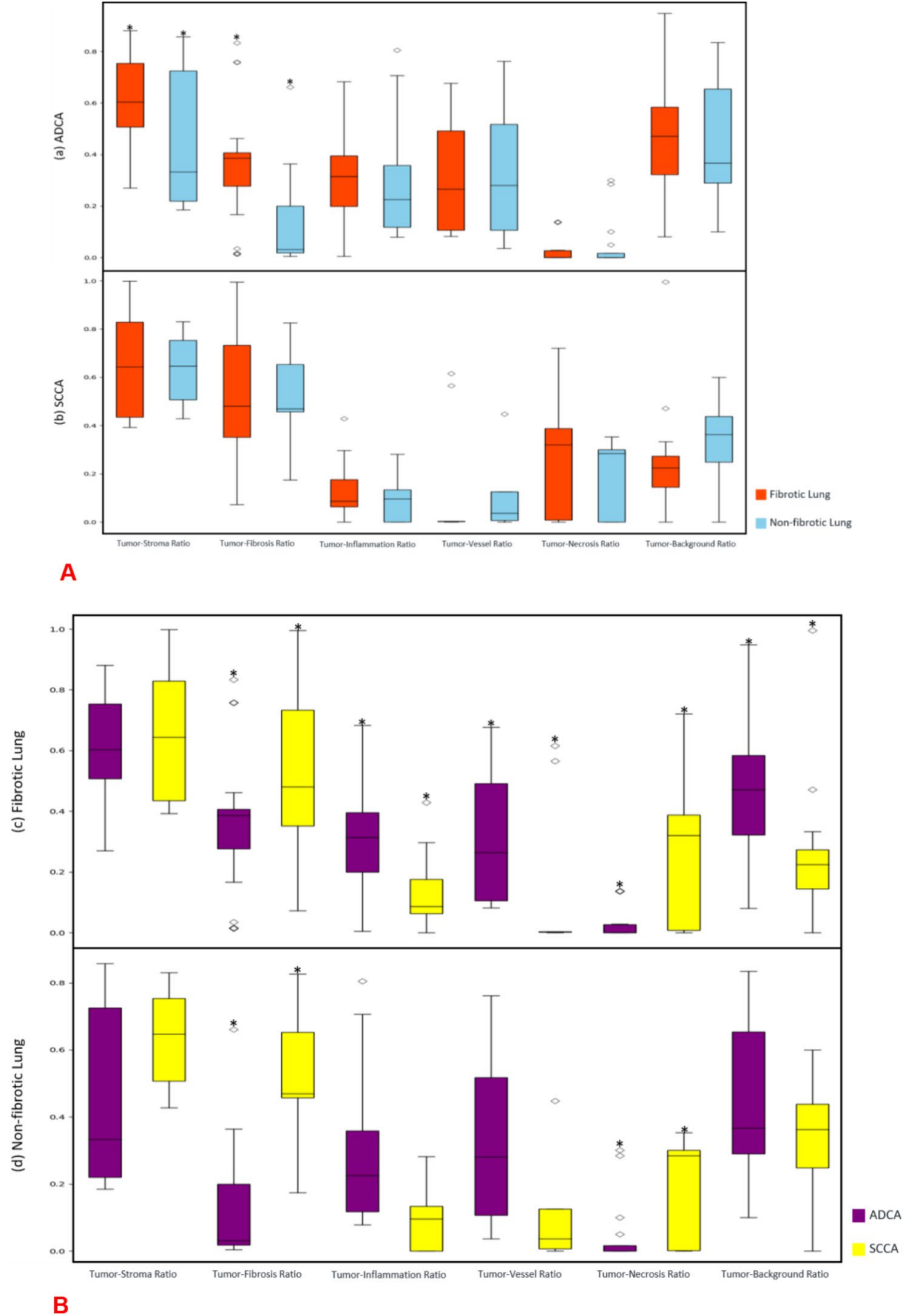
Tumor Microenvironment Analysis (TME) Analysis: **A**) The box-and-whisker diagram of the tumor microenvironment (TME). In Figure A, (**a**) adenocarcinomas (ADCA) and (**b**) squamous cell carcinomas (SCCA) are analyzed separately in fibrotic and non-fibrotic lung. **B**) ADCA and SCCA are evaluated together in (**c**) fibrotic and (**d**) non-fibrotic lung environments. The edges of the boxes correspond to the 25th and 75th percentiles, and the length of the whiskers is 1.5 times the interquartile range. Outliers beyond this limit are shown in hollow blocks. Statistically significant differences are marked with asterisk (p-value *≤* 0.05)

Two analyses were performed. First, each cancer subtype was evaluated separately. For ADCA, there were statistically significant differences in TSR and TFR between fibrotic and non-fibrotic lungs (p-value < 0.005), where TSR and TFR in fibrosis were 28% and 32% greater, respectively. (Fig. 5 and Supplementary Tables 1A and 1B) For SCCA, no significant differences were noted in any TME metrics.

In the second analysis, both ADCA and SCCA were analyzed together within the (1) fibrotic and (2) non-fibrotic backgrounds. In a fibrotic lung environment, ADCA and SCCA exhibited significant differences in TIR (ADCA 30.4%, SCCA 13.2%) and TVR (ADCA 32.3%, SCCA 13.3%), but no differences in TSR (ADCA 67.1%, SCCA 54.1%), or TFR (ADCA 44.8%, SCCA 47.2%), indicating similar fibrosis percentage in both. In contrast, within the non-fibrotic environment, SCCA had a 39% higher TFR compared to ADCA. No significant differences were noted in other TME ratios.

### Correlation with doubling times

A comparative sub-analysis using DT was performed on 21 cases, including 12 ADCA and 9 SCCA in ***fibrotic*** lung with > 2 available CT scans.

The ADCA were divided into two groups based on the median DT (139 days): short DT (20–94) and long DT (139–1001). The SCCA were not stratified because of a narrower DT range (17–297 days with a normal distribution).

**Fig. 6:**
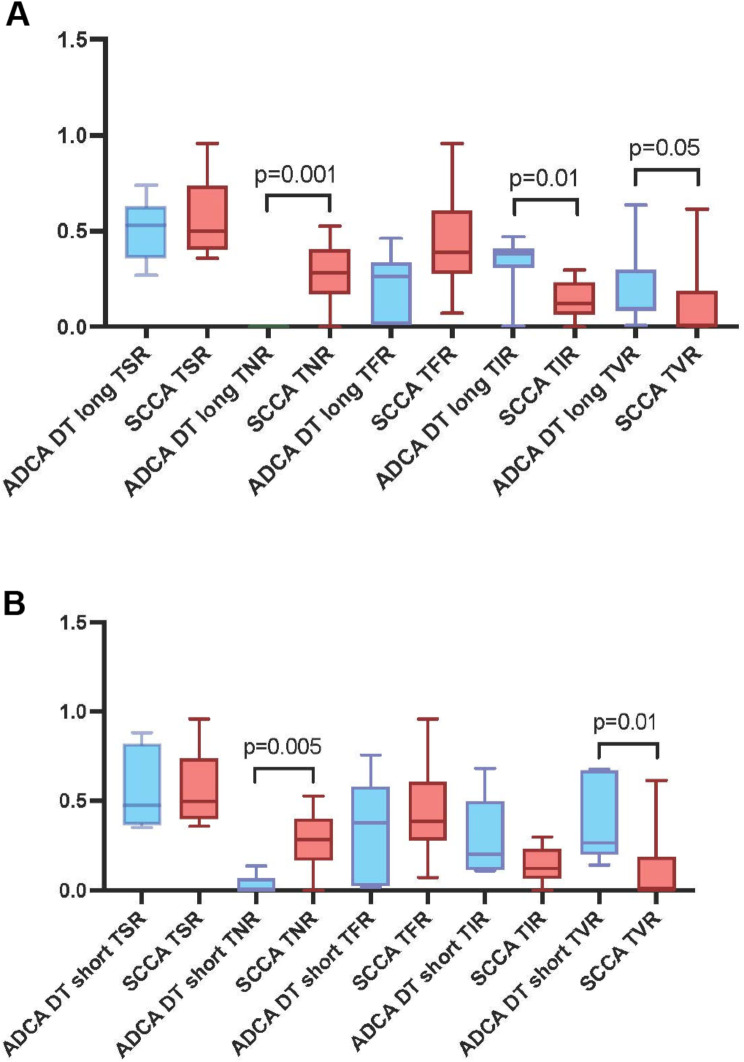
Doubling times in fibrotic lung: (**A**) Comparison between adenocarcinoma with long doubling time and squamous cell carcinoma showing statistically significant differences between tumor necrosis ratio, tumor inflammation ratio and tumor vessel ratio. (**B**) Comparison between adenocarcinoma with short doubling time and squamous cell carcinoma showing statistically significant differences between tumor necrosis ratio and tumor vessel ratio. ADCA: Adenocarcinoma, SCCA: Squamous Cell Carcinoma, DT: Doubling Time, TSR: Tumor Stroma Ratio, TNR: Tumor Necrosis Ratio, TFR: Tumor Fibrosis Ratio, TIR: Tumor Inflammation Ratio, TVR: Tumor Vessels Ratio

First, there were differences in median DT: ADCA = 139 days and SCCA = 72 days. Second, there were statistically significant differences between ADCA long DT and SCCA in TNR (*p* = 0.001), TIR (*p* = 0.013) and TVR (*p* = 0.05). (Fig. 6 and Supplementary Table 2) Third, there were statistically significant differences between the ADCA short DT and SCCA in TNR (*p* = 0.005) and TVR (*p* = 0.01).

The DT of non-fibrotic cases was limited (2 SCCA and 5 ADCA cases in total), which constrained further comparative analysis.

## Discussion

This study utilized H&E WSI (1) to develop a combined EITL—RF multiclass segmentation AI model and (2) evaluated the TME of ADCA and SCCA in fibrotic and non-fibrotic lung backgrounds.

### Artificial intelligence model

Several pathology studies have previously used WSI and CNN, including to evaluate lung cancers [16] and applied a RF algorithm [29, 30]. A limited number have separately described the utility of EITL [31] or human-in-the-loop [32]. To our knowledge, this study is novel, because it is the first to describe an interactive combined EITL—RF—CNN model.

The combined EITL—RF—CNN model addresses current challenges. Typically, CNN use large training sets (hundreds to thousands of WSI) to obtain high accuracy and meaningful results [33]. Moreover, assessment of multiple classification categories requires even greater input data for model training and validation [33]. While the availability of limited data sets can be overcome by annotations, this is time-consuming and requires expertise; as a result, slides are either only partially annotated for training or not at all.

A two-step auto-segmentation process, as described in this study, with (1) supervised review and revise EITL—RF model for slide annotation and (2) CNN can evaluate multiple categories with limited data sets (25 slides from 13 cases in this study) and yield high performance metrics, save time and decrease costs. In contrast, unsupervised learning models necessitate vast amounts of data that may require years to acquire, be unavailable, or demand significant computing systems.

The EITL [32, 34] iterative annotation mechanism enables pathologists to interact with lightweight (e.g., RF) machine-learning models and create fully annotated slides with only partial slide annotations as input by a pathologist. With an EITL—RF model, the training data size can be drastically expanded with smaller number of H&E WSI, and the quality of annotations also optimized through iterations.

Second, most deep learning models of lung cancer using WSI have evaluated a single parameter, like presence/absence of carcinoma or subclassification of carcinoma (e.g. ADCA versus SCCA) and yield area under the curve (AUC) of approximately 0.9 [16]. Meanwhile, 0.6 to 0.8 AUC is the range of more complex tasks [16]. We examined multiple categories and achieved performance metrics expected for single parameter analysis.

The current study evaluated more parameters than previously described and compared the TME within fibrotic and non-fibrotic lung. This model provides multiple significant advantages. First, it uses only H&E-stained slides and no special ancillary studies (e.g., multiple antibodies) or equipment (e.g., immunofluorescence microscope). Second, it provides quantitative analysis. Third, the analysis is performed on all slides to capture heterogeneity. In contrast, a representative slide(s) is used to extrapolate global results when performing immunohistochemical or multiplex immunofluorescence analyses.

### Tumor microenvironment

Our analysis demonstrates notable differences in the TME of fibrotic and non-fibrotic lung. In the ***first analysis*** of ADCA and SCCA, there are significant differences in ADCA TSR and TFR (both greater in the fibrotic compared to non-fibrotic lung). Meanwhile, no differences were noted between the fibrotic and non-fibrotic SCCA cohort. In the ***second analysis*** of fibrotic and non-fibrotic lung, SCCA demonstrated significantly greater TFR and significantly less TIR and TVR compared to ADCA.

To the best of our knowledge, only a single study has examined the TME of lung cancer using WSI-CNN [35], and a single study has applied CNN for tumor micro-vessel assessment [36]. No reports have compared TME in fibrotic vs. non-fibrotic lung or concomitantly analyzed individual stromal components (i.e., fibrosis, inflammation, vessels). In their study of the TME, Wang et al. evaluated > 900 ADCA and > 1900 WSI to generate a spatial map of ADCA and its TME (i.e., stromal cells and lymphocytes) using CNN and achieved an overall 90.1% accuracy in the testing dataset [35]. Our model had similar performance metrics and evaluated both ADCA and SCCA. In their study of 88 lung ADCA, Yi et al. used CNN to evaluate the tumor micro-vessels [36]. Meanwhile, our model represents the first to evaluate tumor micro-vessels amidst additional components of the TME using CNN.

There were notable differences in TFR, TNR, TIR, and TVR between ADCA and SCCA in fibrotic lung. Based on these findings, one may hypothesize that fibrosis is an increased risk factor for developing SCCA. Moreover, using DT as a surrogate for prognosis [37], increased TIR and TVR may be associated with less aggressive behavior due to greater accessibility of immune cells through relatively increased vasculature.

### Limitations

Our study had several limitations that should be acknowledged. Firstly, the fibrotic and non-fibrotic cohorts were defined based on CT scans rather than through interdisciplinary clinical, radiological and pathological correlation. Second, only biopsies were available for the non-fibrotic SCCA cohort. Third, the model evaluated only ADCA and SCCA and its performance may vary across various histological subtypes. Accurately differentiating lepidic adenocarcinoma from reactive cells may present difficulties for the model. These limitations highlight the need for further research and refinement of the model to address specific challenges and expand its applicability to a broader range of tumor types and histological features. Additionally, the cohort of fibrotic TME was derived from multiple etiologies, and DT rather than overall survival data were used.

### Future directions

Future steps include separately evaluating and comparing results between biopsies and resections to determine if biopsies could serve as surrogates for larger specimens, both for purposes of training and predicting other data, such as outcomes. Additional prospective analyses include evaluation at a patient-level and post-processing to evaluate spatial relationships among various parameters and application of the model to extra-institutional datasets for reproducibility studies. The correlation of TSR, TFR, TIR and TVR with outcomes—a likely more robust parameter than DT—is required to determine if these parameters impact prognoses and should be incorporated into routine pathological assessment. Moreover, an investigation comparing results of pathologist and model assessments of complete or major pathological response in ADCA or SCCA resected following neoadjuvant therapies would determine feasibility in clinical trials that may potentially provide expeditious results and reduced costs. Finally, the current study represents a subset derived from previously studied carcinomas in fibrotic and non-fibrotic lung. The cases were evaluated by a radiologist on chest CT scans and subsequently classified on CT scans using CNN [18, 27]. With overlapping results among the former and current analyses, these studies provide a platform for launching a collective radiology, pathology and AI approach with potential synergies in diagnoses, management and prognosis.

## Conclusion

In conclusion, this study introduces an innovative approach by combining EITL—RF and CNN to augment WSI analysis and represents the first AI lung cancer model to evaluate multiple features that detail the TME of ADCA and SCCA in fibrotic and non-fibrotic lung. The integration of this comprehensive deep learning model with radiology, molecular diagnostic and clinical outcomes has the potential to provide a more objective and multi-dimensional assessment of lung cancer. By incorporating these analyses, predictive and prognostic information can be enhanced, leading to improved management strategies for patients.

### Electronic supplementary material

Below is the link to the electronic supplementary material.

Supplementary Material 1
