# Supplementary material for: Combined expert-in-the-loop—random forest multiclass segmentation U-net based artificial intelligence model: evaluation of non-small cell lung cancer in fibrotic and non-fibrotic microenvironments
Source: J Transl Med. 2024 Jul 8;22:640. doi: 10.1186/s12967-024-05394-2 (PMC11232199; doi:10.1186/s12967-024-05394-2)
Supplement: Supplementary file 1 — Supplementary Material 1 [file 12967_2024_5394_MOESM1_ESM.docx]

**Supplementary Figures & Legends**


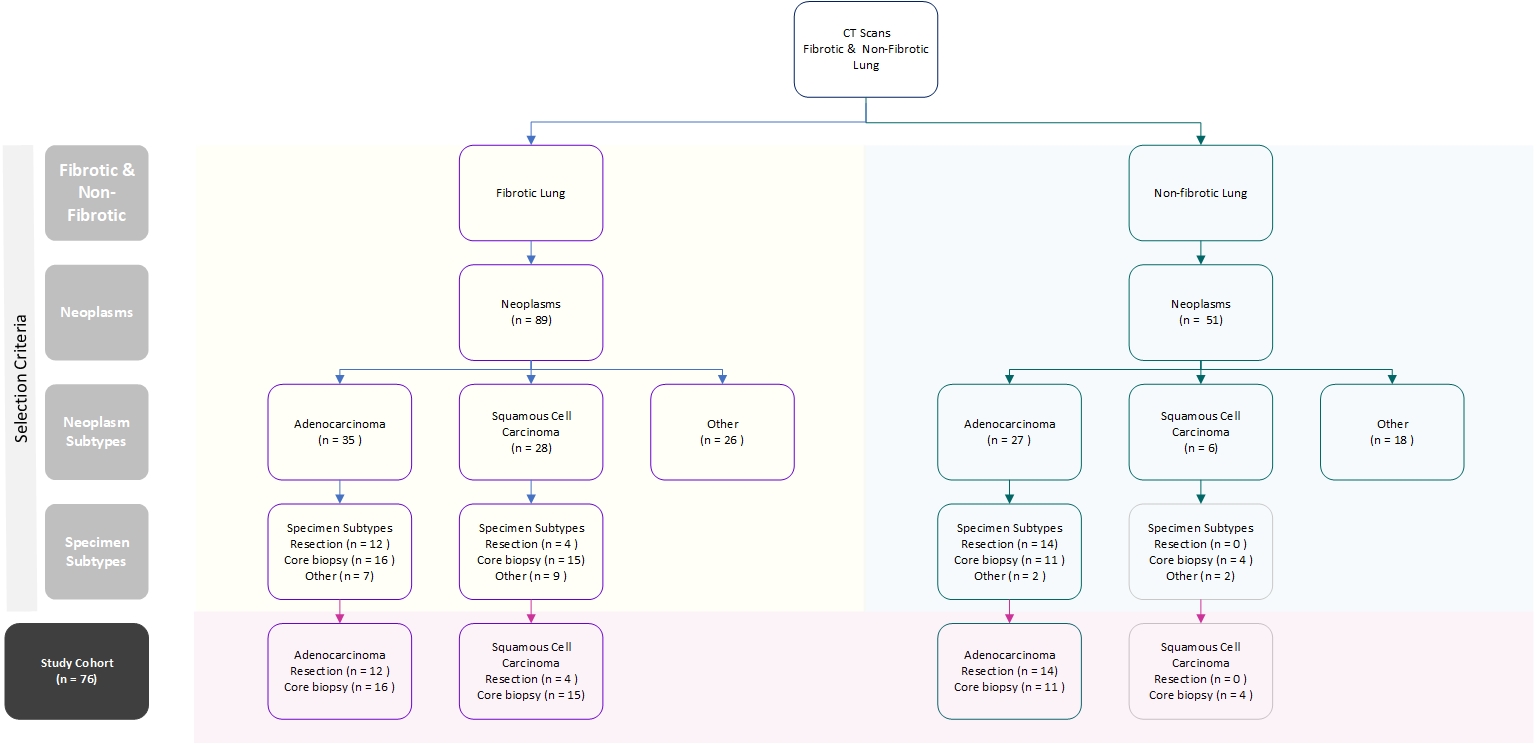


**Supplementary Figure 1**: Study cohort of nodules within fibrotic and non-fibrotic lung

| TME metrics | Cancer types | Lung environment | Average | 95% CI | P-value |
| --- | --- | --- | --- | --- | --- |
| TSR | Adenocarcinoma | Fibrotic | 0.5967 | [0.5229 – 0.6704] | 0.0405* |
|  |  | Non-fibrotic | 0.4532 | [0.3222 – 0.5843] |  |
|  | Squamous | Fibrotic | 0.6461 | [0.5002 – 0.7919] | 0.9046 |
|  |  | Non-fibrotic | 0.6337 | [0.4637 – 0.8036] |  |
| TFR | Adenocarcinoma | Fibrotic | 0.3589 | [0.2628 – 0.4550] | 0.0013* |
|  |  | Non-fibrotic | 0.1308 | [0.0385 – 0.2331] |  |
|  | Squamous | Fibrotic | 0.5483 | [0.3626 – 0.7342] | 0.8296 |
|  |  | Non-fibrotic | 0.5158 | [0.2137 – 0.8179] |  |
| TIR | Adenocarcinoma | Fibrotic | 0.3039 | [0.2281 – 0.3794] | 0.8477 |
|  |  | Non-fibrotic | 0.2910 | [0.1602 – 0.4218] |  |
|  | Squamous | Fibrotic | 0.1316 | [0.0519 – 0.2117] | 0.6617 |
|  |  | Non-fibrotic | 0.1023 | [-0.0422 – 0.2468] |  |
| TVR | Adenocarcinoma | Fibrotic | 0.3235 | [0.2087 – 0.4382] | 0.9306 |
|  |  | Non-fibrotic | 0.3312 | [0.1716 – 0.4988] |  |
|  | Squamous | Fibrotic | 0.1325 | [-0.0670 – 0.3320] | 0.9470 |
|  |  | Non-fibrotic | 0.1235 | [-0.1099 – 0.3568] |  |
| TNR | Adenocarcinoma | Fibrotic | 0.0249 | [0.0042 – 0.0457] | 0.4182 |
|  |  | Non-fibrotic | 0.0446 | [-0.0056 – 0.0942] |  |
|  | Squamous | Fibrotic | 0.2761 | [0.1263 – 0.4258] | 0.4652 |
|  |  | Non-fibrotic | 0.1880 | [-0.0269 – 0.4029] |  |
| TBR | Adenocarcinoma | Fibrotic | 0.4861 | [0.3763 – 0.5960] | 0.6310 |
|  |  | Non-fibrotic | 0.4466 | [0.3122 – 0.5818] |  |
|  | Squamous | Fibrotic | 0.2641 | [0.0950 – 0.4330] | 0.6348 |
|  |  | Non-fibrotic | 0.3300 | [0.0512 – 0.6087] |  |

**Supplementary Table 1A**

| TME metrics | Lung environment | Cancer types | Average | 95% CI | P-value |
| --- | --- | --- | --- | --- | --- |
| TSR | Fibrotic | Adenocarcinoma | 0.5967 | [0.5229 – 0.6704] | 0.4734 |
|  |  | Squamous | 0.6461 | [0.5002 – 0.7918] |  |
|  | Non-fibrotic | Adenocarcinoma | 0.4532 | [0.3221 – 0.5843] | 0.1224 |
|  |  | Squamous | 0.6337 | [0.4637 – 0.8036] |  |
| TFR | Fibrotic | Adenocarcinoma | 0.3589 | [0.2628 – 0.4550] | 0.0392* |
|  |  | Squamous | 0.5483 | [0.3626 – 0.7342] |  |
|  | Non-fibrotic | Adenocarcinoma | 0.1308 | [0.0384 – 0.2231] | 0.0009* |
|  |  | Squamous | 0.5158 | [0.2137 – 0.8179] |  |
| TIR | Fibrotic | Adenocarcinoma | 0.3039 | [0.2281 – 0.3794] | 0.0032* |
|  |  | Squamous | 0.1316 | [0.0515 – 0.2117] |  |
|  | Non-fibrotic | Adenocarcinoma | 0.2911 | [0.1609 – 0.4218] | 0.0967 |
|  |  | Squamous | 0.1023 | [-0.0422 – 0.2468] |  |
| TVR | Fibrotic | Adenocarcinoma | 0.3235 | [0.2087 – 0.4382] | 0.0413* |
|  |  | Squamous | 0.1325 | [-0.0670 – 0.3320] |  |
|  | Non-fibrotic | Adenocarcinoma | 0.3312 | [0.1716 – 0.4908] | 0.1189 |
|  |  | Squamous | 0.1235 | [-0.1099 – 0.3568] |  |
| TNR | Fibrotic | Adenocarcinoma | 0.0249 | [0.0042 – 0.0457] | 0.0003* |
|  |  | Squamous | 0.2760 | [0.1269 – 0.4258] |  |
|  | Non-fibrotic | Adenocarcinoma | 0.0443 | [-0.0056 – 0.0941] | 0.0247* |
|  |  | Squamous | 0.1880 | [-0.0269 – 0.4029] |  |
| TBR | Fibrotic | Adenocarcinoma | 0.4861 | [0.3763 – 0.5960] | 0.0200* |
|  |  | Squamous | 0.2640 | [0.0950 – 0.4330] |  |
|  | Non-fibrotic | Adenocarcinoma | 0.4466 | [0.3121 – 0.5810] | 0.3679 |
|  |  | Squamous | 0.3300 | [0.0512 – 0.6087] |  |

**Supplementary Table 1B**

**A: Doubling Time in Fibrotic Lung: Adenocarcinoma Long Doubling Time vs Squamous Cell Carcinoma**

|  | **TSR** | **TNR** | **TFR** | **TIR** | **TVR** | **DT** |
| --- | --- | --- | --- | --- | --- | --- |
| **ADCA Long DT**  **(n=7)** | 0.51  ± 0.17 | 0.00003  (0-0.0004) | 0.22  (0.01-0.34) | 0.38  (0.31-0.41) | 0.1  (0.84-0.30) | 315  (265-520) |
| **SCCA (n=9)** | 0.57  ± 0.21 | 0.28  (0.23-0.37) | 0.44  (0.31-0.53) | 0.12  (0.73-0.20) | 0.004  (0.002-0.073) | 72  (55-128) |
| **P Value** | 0.53 | 0.001 | 0.07 | 0.013 | 0.05 | <0.001 |

**B: Doubling Time in Fibrotic Lung: Adenocarcinoma Short Doubling Time vs Squamous Cell Carcinoma**

|  | **TSR** | **TNR** | **TFR** | **TIR** | **TVR** | **DT** |
| --- | --- | --- | --- | --- | --- | --- |
| **ADCA Short DT (n=6)** | 0.60  ± 0.22 | 0.00023  (0-0.005) | 0.39  (0.34-0.76) | 0.26  (0.12-0.43) | 0.38  (0.14-0.67) | 42  (27-87) |
| **SCCA (n=9)** | 0.57 ± 0.21 | 0.28  (0.23-0.37) | 0.44  (0.31-0.53) | 0.12  (0.07-0.19) | 0.004  (0.002-0.073) | 72  (55-128) |
| **P Value** | 0.74 | 0.005 | 0.76 | 0.06 | 0.01 | 0.02 |

**Supplementary Tables 2A and 2B:** Sub-analysis Doubling Time in Fibrotic Lung

Values are mean ± standard deviation, or median (inter-quartile range).

ADCA: Adenocarcinoma, SCCA: Squamous Cell Carcinoma, DT: Doubling Time, TSR: Tumor Stroma Ratio, TNR: Tumor Necrosis Ratio, TFR: Tumor Fibrosis Ratio, TIR: Tumor Inflammation Ratio, TVR: Tumor Vessels Ratio
